# Inhibition of Chk1 with the small molecule inhibitor V158411 induces DNA damage and cell death in an unperturbed S-phase

**DOI:** 10.18632/oncotarget.13119

**Published:** 2016-11-04

**Authors:** Joanne Wayne, Teresa Brooks, Andrew J. Massey

**Affiliations:** ^1^ Vernalis Research, Cambridge, CB21 6GB, UK

**Keywords:** Chk1, kinase inhibitor, replication stress, H2AX, DNA damage

## Abstract

Chk1 kinase is a critical component of the DNA damage response checkpoint and Chk1 inhibitors are currently under clinical investigation. Chk1 suppresses oncogene-induced replication stress with Chk1 inhibitors demonstrating activity as a monotherapy in numerous cancer types. Understanding the mechanism by which Chk1 inhibitors induce DNA damage and cancer cell death is essential for their future clinical development. Here we characterize the mechanism by which the novel Chk1 inhibitor (V158411) increased DNA damage and cell death in models of human cancer. V158411 induced a time- and concentration-dependent increase in γH2AX-positive nuclei that was restricted to cells actively undergoing DNA synthesis. γH2AX induction was an early event and correlated with activation of the ATR/ATM/DNA-PKcs DNA damage response pathways. The appearance of γH2AX positive nuclei preceded ssDNA appearance and RPA exhaustion. Complete and sustained inhibition of Chk1 kinase was necessary to activate a robust γH2AX induction and growth inhibition. Chk1 inhibitor cytotoxicity correlated with induction of DNA damage with cells undergoing apoptosis, mitotic slippage and DNA damage-induced permanent cell cycle arrest. We identified two distinct classes of Chk1 inhibitors: those that induced a strong increase in γH2AX, pChk1 (S317) and pRPA32 (S4/S8) (including V158411, LY2603618 and ARRY-1A) and those that did not (including MK-8776 and GNE-900). Tumor cell death, induced through increased DNA damage, coupled with abrogation of cell cycle checkpoints makes selective inhibitors of Chk1 a potentially useful therapeutic treatment for multiple human cancers.

## INTRODUCTION

The human genome experiences environmentally and endogenously generated DNA damage on a daily basis. The DNA damage response (DDR) pathway, a cooperation of complex DNA repair and cell cycle control pathways of which ATR and Chk1 are central components, has evolved to help cells manage this DNA damage burden. Oncogene activation and/or the loss of tumor suppressor proteins drive tumor cell proliferation resulting in increased replication stress. In pre-cancerous lesions, increased replication stress is an important contributor to genome instability resulting in the increased acquisition of genomic changes and cancer development [1-3]. The uncontrolled replication burst that occurs in tumor cells following dysregulation of cell cycle controls results in replication fork stalling and DNA double-strand breaks (DSBs) arising from fork collapse [4-6]. Sustained activation of the DDR in precancerous lesions leads to apoptosis and/or senescence preventing cancer development [7-9]. The acquisition of additional mutations allows cells to evade these checkpoints resulting in transformation and carcinogenesis.

ATR and Chk1 kinases, key components of the S-phase checkpoint, are critical for the cellular response to replication stress [10-12]. Following replication fork stalling, the replicative helicase continues to unwind DNA in front of the stalled polymerase. The subsequent generation of long runs of ssDNA is bound by RPA. Activation of ATR assembled on the RPA-bound ssDNA by TOPBP1 results in Chk1 phosphorylation on S317 and S345 [13, 14] followed by *cis*-autophosphorylation on S296 [13]. Active Chk1 consequently stabilizes replication forks, prevents cleavage by Mus81-Eme1-Mre11 nucleases [14, 15], activates cell cycle arrest (through control of Cdc25 phosphatase stability) [16-18], prevents new origin firing [19, 20] and activates homologous recombination repair [21]. Replication can restart once the source of arrest has either been bypassed or repaired.

Numerous inhibitors of Chk1 are in pre-clinical and clinical development with the focus predominantly on their ability to potentiate the cytotoxicity of genotoxic chemotherapy drugs or ionizing radiation. Genotoxic stress induced by these agents induces the DDR. Abrogation of cell cycle arrest by a Chk1 inhibitor allows progress into mitosis with high levels of DNA damage and subsequent cell death, especially in p53-defective cancer cells [22]. A range of Phase I and II clinical trials are currently evaluating this approach [23, 24]. Suppressing the DNA damage response through the inhibition of Chk1 and/or ATR has the potential to increase DNA damage and cell death in a tumor cell selective fashion. A range of cancer cell lines [25-29] and genetically engineered tumor models [30, 31] have exhibited hyper-sensitivity to Chk1 inhibitor monotherapy. These models feature either increased replicative stress or DNA repair pathway defects. V158411 is a potent, selective inhibitor of Chk1 and demonstrates activity both as a monotherapy and in combination with a range of cytotoxic chemotherapeutic agents [32-35]. Understanding the temporal and spatial tumor cell response to V158411 monotherapy is critical for further clinical development. Here we utilized single cell immunofluorescent high content microscopy and live cell imaging to characterize the induction of DNA damage and the mode of cell death in tumor cells treated with V158411.

## RESULTS

### Chk1 inhibitor-induced DNA damage is restricted to predominantly S-phase cells

**Figure 1:**
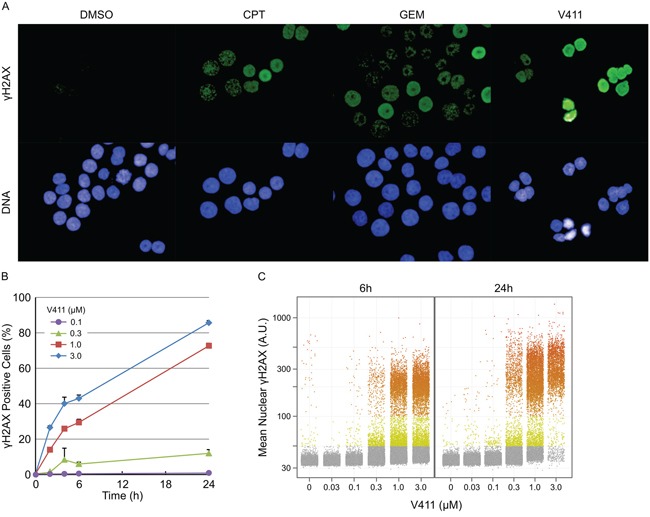
Inhibition of Chk1 induces DNA damage in a dose- and time-dependent fashion **A.** Example images of γH2AX staining in HT29 cells following 24 h treatment with 1 μM V411, 0.1 μM camptothecin (CPT) or 0.1 μM gemcitabine (GEM). HT29 cells were treated with the indicated concentrations of V411 for 0 to 24 h and the number of cells staining positive for **B.** and the average nuclear intensity of **C.** γH2AX determined by single cell immunofluorescent imaging (n=4, mean ± SD).

**Figure 2:**
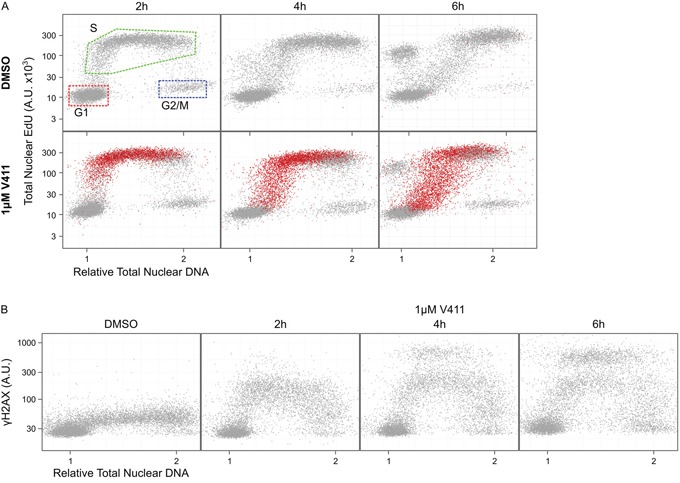
DNA damage induced by Chk1 inhibition is restricted to S-phase cells HT29 cells were incubated with EdU for 15 minutes to label S-phase cells then 1 μM V411 for 2 to 6 h. Total nuclear intensity of indicated fluorescent markers was determined by single cell immunofluorescent imaging. **A.** Plots of total nuclear DNA versus total nuclear EdU for γH2AX negative (grey) versus γH2AX positive (red) cells. **B.** Plots show total nuclear DNA versus average nuclear γH2AX.

Chk1 inhibition by V158411, in HT29 cells, increased the fraction of cells staining positive for pan-nuclear γH2AX, a marker of DNA double strand breaks (DSBs), rather than γH2AX foci as was observed with the DNA damaging agents gemcitabine and camptothecin (Figure 1A). This increase in γH2AX was dose and time dependent (Figure 1B and 1C) with the γH2AX intensity in the positive nuclei increasing with time. Co-staining with EdU, a marker of active DNA synthesis, indicated that V158411-induced DNA damage was restricted to actively proliferating S-phase cells (Figure 2A). A comparison of γH2AX intensity to total nuclear DNA identified the γH2AX-positive cells being located in S-phase (Figure 2B and Supplementary Figure S1).

### Chk1 inhibition activates the ATR/ATM/DNA-PKcs DDR pathways

**Figure 3:**
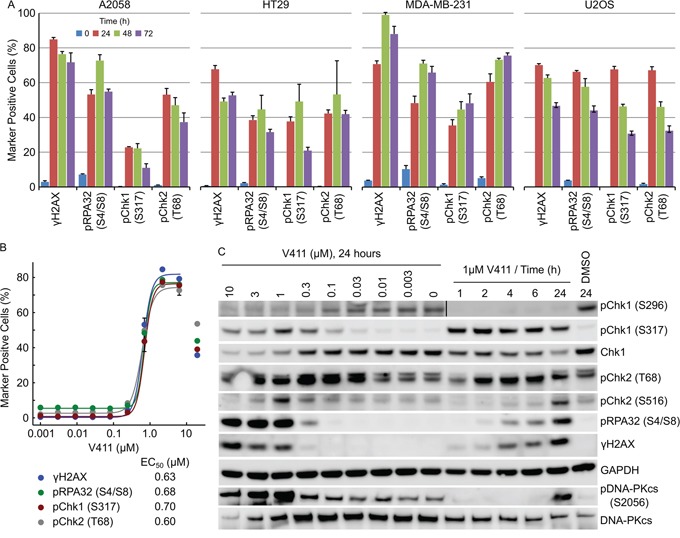
Chk1 inhibition activates the ATR/ATM/DNA-PKcs DNA damage response pathways **A.** Cell lines were treated with 1 μM V411 for the indicated times. The fraction of γH2AX, pRPA32 (S4/S8), pChk1 (S317) or pChk2 (T68) positive nuclei were determined by single cell immunofluorescent imaging (n=4, mean ± SD). **B.** HT29 cells were treated with increasing concentrations of V411 for 24 h and the fraction of positive nuclei determined as above. **C.** HT29 cells were treated with the indicated concentrations and timings of V411. Cell lysates were immunoblotted with the indicated antibodies.

Treatment of A2058, HT29, MDA-MB-231 or U2OS cells for 24 to 72 hours with V158411 increased the number of nuclei with elevated γH2AX, pChk1 (S317) (an ATR substrate), pChk2 (T68) (an ATM substrate) or pRPA32 (S4/S8) (a DNA-PKcs substrate) staining (Figure 3A). The tumor entity and known oncogenic drivers of the cell lines used are summarized in Supplementary Table S1 and the DNA repair gene status in Supplementary Table S2. In untreated cells, increased pRPA32 (S4/S8) staining was associated with mitosis as detected by co-staining with pHH3 (S10) (Supplementary Figure S2A). The concentration of V158411 required to half-maximally increase the number of HT29 marker positive cells was approximately equal for all four markers (Figure 3B).

### γH2AX induction is an early event following Chk1 inhibition and occurs prior to ssDNA appearance

**Figure 4:**
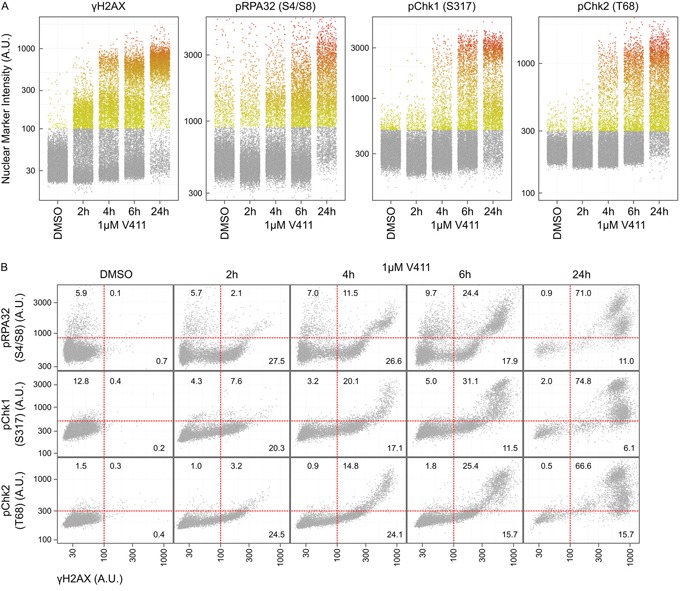
Phosphorylation of H2AX on Serine 139 is an early event following Chk1 inhibition and occurs concurrently with downstream DDR signaling **A.** The time-dependent increase in average nuclear γH2AX, pRPA32 (S4/S8), pChk1 (S317) and pChk2 (T68) intensity in HT29 cells following treatment with 1 μM V411 was determined by single cell immunofluorescent imaging. **B.** Co-expression analysis of γH2AX with pRPA32 (S4/S8), pChk1 (S317) and pChk2 (T68) in HT29 cells. The numbers indicate the fraction of positive cells in each quadrant.

γH2AX positive nuclei appeared rapidly, within 2 hours, following Chk1 inhibition in HT29 or U2OS cells (Figures 3C and 4A and Supplementary Figure S3A). Increased pChk1 (S317) and pChk2 (T68) could be detected within 1 and 2 hours respectively following V158411 treatment (Figure 3C and Supplementary Figures S2B and S2C) but the detection of positive nuclei by immunofluorescence was delayed with positive nuclei taking an extra 2 to 4 hours to appear (Figure 4A and Supplementary Figure S3A). pRPA32 (S4/S8) had the longest delay taking around 4 and 6 hours (post Chk1 inhibition) to appear in U2OS and HT29 cells respectively. In all four cell lines, γH2AX expression was tightly associated with RPA32, Chk1 and Chk2 phosphorylation with the majority of γH2AX-positive cells staining positive for pRPA32 (S4/S8), pChk1 (S317) and pChk2 (T68) 24 hours after V158411 addition (Figure 4B and Supplementary Figures S3B and S4).

**Figure 5:**
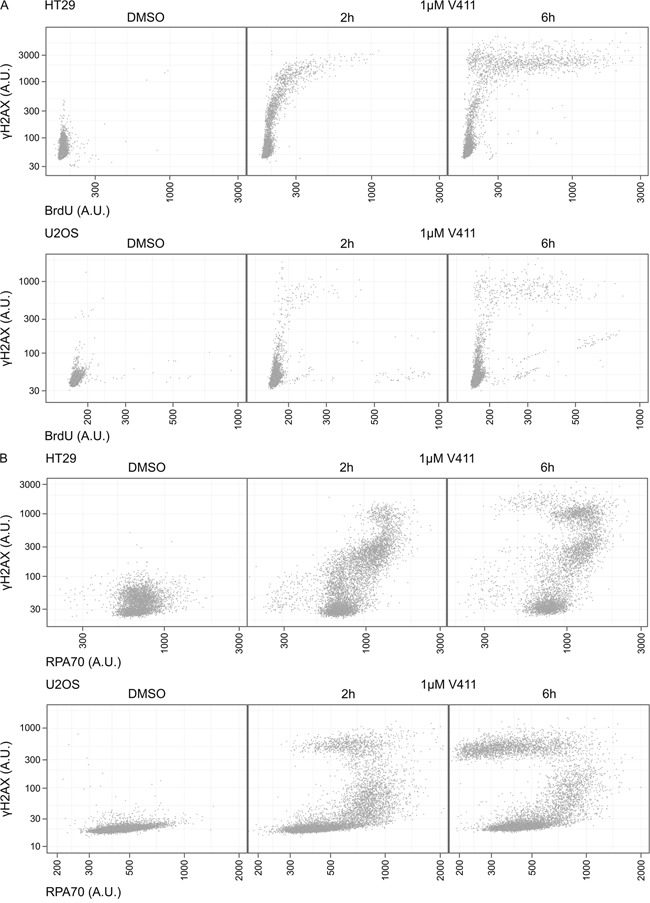
Chk1 inhibition induces γH2AX prior to depletion of the available pool of RPA or the appearance of ssDNA **A.** HT29 or U2OS cells were labelled with BrdU for 42 h then 1 μM V411 for 2 or 6 h, or DMSO for 6 h. ssDNA was identified with an anti-BrdU antibody under non-denaturing conditions. **B.** HT29 or U2OS cells were treated with 1 μM V411 for 2 or 6 h and mean nuclear γH2AX or RPA70 determined by single cell immunofluorescent analysis.

ssDNA in cells can be visualized by labelling cells with BrdU and analyzing BrdU staining under non-denaturing conditions. In HT29 and U2OS cells, γH2AX staining was detected within 2 hours of V158411 treatment but longer treatment (6 hours) with V158411 was required before ssDNA could be reliably detected (Figure 5A). Increased ssDNA was associated with intense γH2AX staining. Unscheduled origin firing due to ATR inhibition results in the generation of large amounts of ssDNA and exhaustion of the pool of RPA. This can be visualized by a time-dependent increase in γH2AX staining intensity without a concomitant increase in chromatin bound RPA [36]. Chk1 inhibition induced γH2AX in cells prior to depletion of the available pool of RPA. γH2AX, without co-localization of additional RPA, appeared at later time points and coincided with increased ssDNA (Figure 5B). The damage formed, following Chk1 inhibitor treatment, was refractory to TUNEL labelling (Supplementary Figure S5).

### Complete and sustained inhibition of Chk1 is necessary to induce a robust cellular response

**Figure 6:**
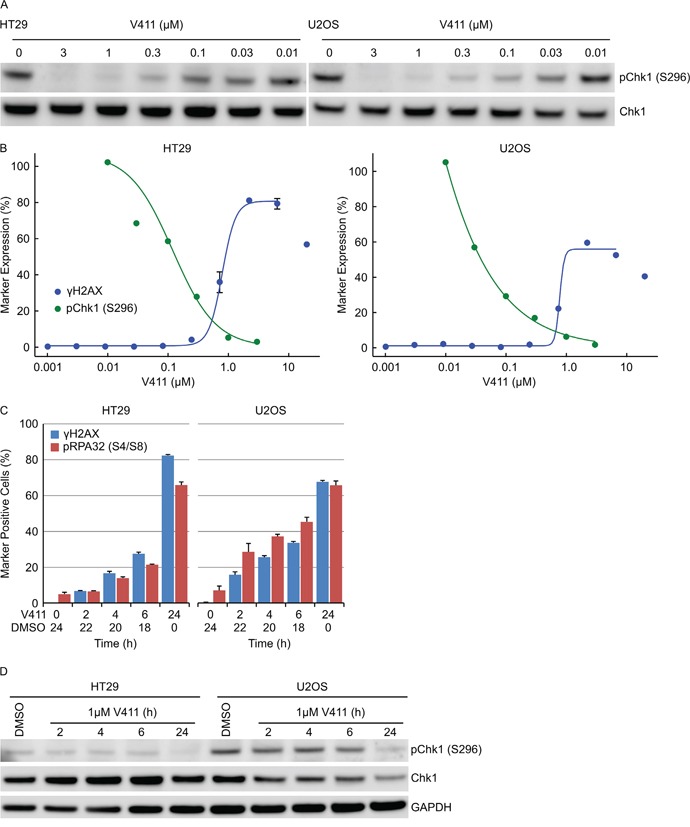
Complete and sustained inhibition of Chk1 is necessary to induce a robust cellular response **A.** HT29 or U2OS cells were treated with indicated concentrations of V411 for 2 h and cell lysates probed with antibodies to pChk1 (S296) and total Chk1. **B.** The relative expression levels of pChk1 (S296) was determined by densitometric analysis of the blots above (green) and plotted against the fraction of γH2AX positive cells following 24 h V411 treatment (blue). **C.** Cells were treated with 1 μM V411 for the indicated times then the V411 media removed, replaced with DMSO containing media and further incubated so that total time in V411-cotaining and DMSO-containing media equaled 24 h. The fraction of γH2AX, pRPA32 (S4/S8), pChk1 (S317) and pChk2 (T68) positive cells were determined by single-cell immunofluorescent imaging (n=4, mean ± SD). **D.** Cells were treated with 1 μM V411 for the indicated times before the V411 containing media was removed, replaced with V411-free media and cells incubated further so that total time in V411-containing and V411-free media equaled 24 h. Cell lysates were immunoblotted with the indicated antibodies.

Chk1 undergoes a *cis* auto-phosphorylation event on serine 296 and is a pharmacodynamic biomarker of Chk1 kinase activity. V158411 induced a dose-dependent decrease in pS296 with an IC_50_ and IC_90_ of 0.12 and 0.77 μM in HT29 cells and 0.039 and 0.59 μM respectively in U2OS cells (Figure 6A and 6B). Almost complete inhibition of Chk1 kinase activity was required before γH2AX positive cells were detected (Figure 6B). EC_50_ values for γH2AX induction were 0.77 and 0.79 μM in HT29 and U2OS cells respectively. In combination with the anti-metabolite gemcitabine, γH2AX nuclei were detected at much lower concentrations of V158411 (EC_50_ 0.017 μM) compared to cells treated with V158411 alone (EC_50_ 0.57 μM, Supplementary Figure S6A). Treatment of HT29 cells with gemcitabine increased pChk1 (S296). Partial inhibition of this increase by V158411 resulted in increased DNA damage (Supplementary Figure S6B). Chk1 inhibition induced DNA damage in cells actively undergoing DNA synthesis only when Chk1 inhibitor was present. Pulse treatment of HT29 or U2OS cells with V158411 for 2, 4 or 6 hours followed by recovery in V158411-free media for 22, 20 or 18 hours respectively resulted in a reduction in the number of cells staining positive for γH2AX or pRPA32 (S4/S8) compared to 24 hour continual treatment (Figure 6C). Chk1 kinase inhibition, following the removal of V158411, was not maintained for the duration of the washout period (Figure 6D) resulting in an attenuated response to Chk1 inhibition.

### Chk1 inhibition induces mitotic failure and DNA damage-induced permanent cell cycle arrest

**Figure 7:**
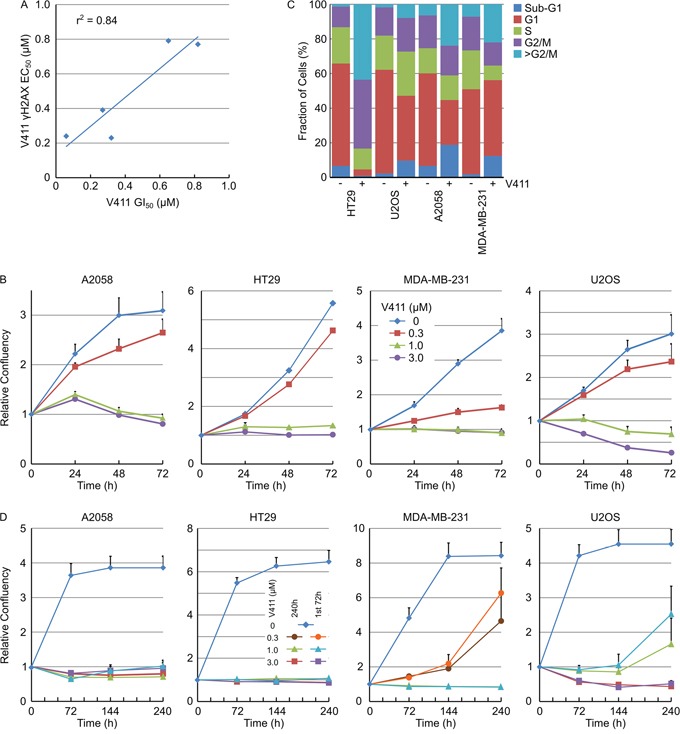
Chk1 inhibitor cytotoxicity correlates with DNA damage and induces mitotic failure and DNA damage-induced permanent cell cycle arrest **A.** Plot of the 72 h GI_50_ versus the 24 h γH2AX induction EC_50_ for 5 cell lines (n ≥ 4). **B.** Cell lines were treated with the indicated concentrations of V411 and cellular confluency determined by repeated live cell imaging (n=6, mean ± SD). **C.** At the 72 h time point, cells treated with 0 or 1 μM V411 were fixed, stained with Hoechst and the cell cycle distribution estimated based on the total nuclear DNA fluorescence by single cell imaging. **D.** Cells were exposed to the indicated concentrations of V411 for the full duration of the experiment (240 h) or for 72 h. After the 72 h treatment, the media was removed, replaced with V411-free media and incubation continued to the end of the experiment. Cellular confluency was determined by repeated live cell imaging (n=6, mean ± SD).

To understand the correlation between γH2AX induction and the effects of Chk1 inhibition on cellular proliferation, the 72 hour GI_50_ value for HT29, U2OS, A2058, MDA-MB-231 and SKOV-3 cells was determined and compared to the γH2AX EC_50_ value. There was a close correlation (r^2^ = 0.84) between DNA damage induction and the anti-proliferative activity of V158411 in this small panel of cell lines (Figure 7A). We utilized daily live cell imaging to understand this further. Using confluency as a measure of cell number (example images for HT29 cells are shown in Supplementary Figure S7A), V158411 induced predominantly cytostasis in HT29 and MDA-MB-231 cells, cytostasis then weak cytotoxicity in A2058 cells and strong cytotoxicity in U2OS cells (Figure 7B). This was confirmed in A2058, MDA-MB-231 and U2OS cells using digital phase imaging to count individual cells (Supplementary Figure S7B). At the end of the 72 hour treatment, the cells were Hoechst stained (Supplementary Figure S7C) and the cell cycle phase determined based on the total DNA content. In HT29, A2058 and MDA-MB-231 cells, 72 hour treatment with V158411 decreased the G1 and S-phase fractions and dramatically increased the number of cells with a DNA content equivalent to G2/M and greater (Figure 7C). This increase in cells with a DNA content >G2/M appeared to be due to a failure of cells to undergo cytokinesis. V158411 did not prevent the entry of cells into mitosis (Supplementary Figure S8). In U2OS cells, the reduction in G1 and increase in G2/M were less marked than the other three cell lines and may reflect the small number of surviving cells available for analysis.

Persistent γH2AX can result in DNA damage-induced cell senescence [37]. γH2AX induction following Chk1 inhibition with V158411 was maintained for at least 72 hours post addition of V158411 (Figure 3A). To determine if V158411-induced growth arrest was reversible, cells were treated with V158411 for 72 hours before the media was removed and replaced with either media containing DMSO or fresh V158411 and cellular growth monitored for a further 168 hours. 72 hour treatment with 1 μM V158411 induced irreversible growth arrest in A2058, HT29 or MDA-MB-231 cells whilst 3 μM was necessary to have an effect on U2OS cells (Figure 7D).

### γH2AX induction and caspase-3 cleavage are mutually exclusive cellular outcomes following Chk1 inhibition

**Figure 8:**
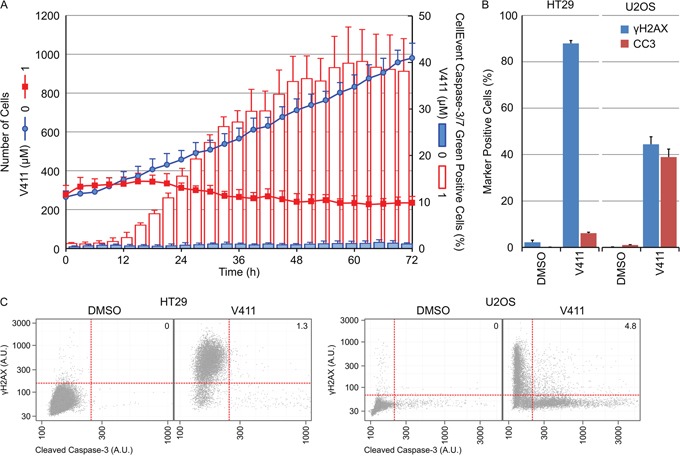
γH2AX induction and caspase-3 cleavage are mutually exclusive cellular outcomes following Chk1 inhibition **A.** U2OS cells were incubated with 0 or 1 μM V411 in the presence of CellEvent Caspase-3/7 Green probe and imaged every 3 hours for 72 h using digital phase imaging to determine cell number and Alexa488 fluorescence to detect cleaved caspase-3/7 positive cells (n=6, mean ± SD). **B.** Following 48 h treatment with 1 μM V411, cells were fixed and γH2AX or cleaved caspase-3 (CC3) expression determined by single cell immunofluorescence analysis (n=4, mean ± SD). **C.** Single cell plots of mean nuclear γH2AX versus CC3 expression. Numbers indicate the percentage of cells staining positive for γH2AX and CC3.

Inhibition of Chk1 in HT29 cells induced predominantly irreversible growth arrest with little cell death whilst in U2OS cells, a significant amount of cell death was apparent (Figure 7B). Live cell imaging was utilized to monitor cell number and the appearance of cleaved capsase-3/7 using a fluorescent caspase substrate. Treatment of U2OS cells with 1 μM V158411 induced a time-dependent decrease in cell number and an increase in caspase-3/7-positive cells. Maximal caspase-dependent apoptosis was observed by 48 hours (Figure 8A). Surprisingly, there was a small fraction of cells that were resistant to V158411-induced cell death (Figures 8A and 7D). U2OS cells express wild-type p53. V158411 generated a weak induction of p53 and p21 in U2OS cells that was reduced compared to the induction by camptothecin or the MDM-2 inhibitor Nutlin 3a (Supplementary Figure S9).

We compared the apoptotic response of U2OS to HT29 cells following Chk1 inhibition. 48 hour treatment of HT29 cells with 1 μM V158411 increased the number of γH2AX positive cells by 86 % but not the number of caspase-3 positive cells. Treatment of U2OS cells with 1 μM V158411 increased the number of γH2AX positive cells by 44 % and caspase-3 positive cells by 39 % (Figure 8B). A comparison of γH2AX versus caspase-3 intensities revealed a dramatic lack of cells staining positive for both γH2AX and cleaved caspase-3 (1.3 % HT29 and 4.8 % U2OS, Figure 8C).

### Various Chk1 inhibitors induce DNA damage and permanent DNA damage-induced cell cycle arrest

**Figure 9:**
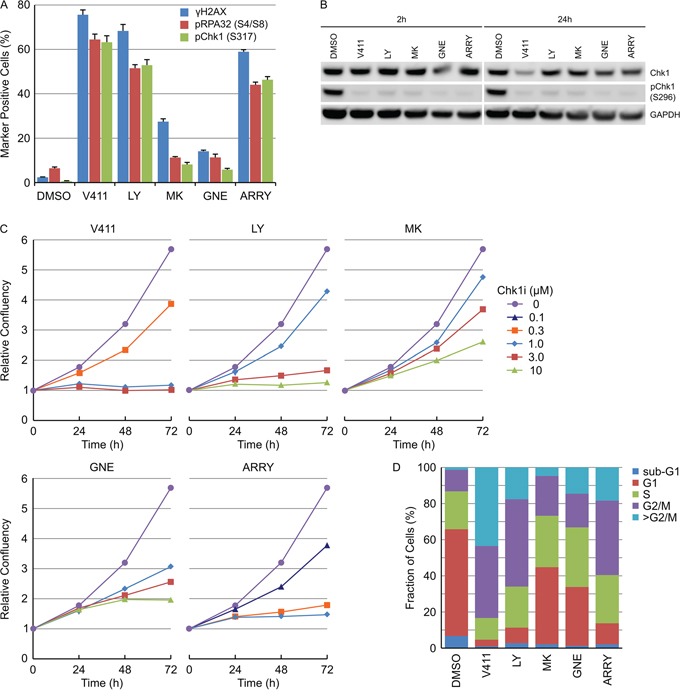
Various Chk1 inhibitors induce DNA damage and permanent DNA damage-induced cell cycle arrest **A.** HT29 cells were treated with 1 μM V411, 3 μM LY2603618 (LY), 3 μM MK-8776 (MK), 3 μM GNE-900 (GNE) or 0.3 μM ARRY-1A (ARRY) for 24 h. The fraction of γH2AX, pRPA32 (S4/S8), pChk1 (S317) or pChk2 (T68) positive nuclei were determined by single cell immunofluorescent imaging (n=4, mean ± SD). **B.** HT29 cells were treated as above for 2 or 24 h. Cell lysates were probed with the indicated antibodies by immunoblotting. **C.** HT29 cellular confluency following treatment with the indicated concentrations of Chk1 inhibitors was determined by repeated live cell imaging (n=3, mean ± SD). **D.** At the 72 h time point, the cells treated with 1 μM V411, 3 μM LY, 3 μM MK, 3 μM GNE or 0.3 μM ARRY were fixed, stained with Hoechst and the cell cycle distribution estimated based on the total nuclear DNA fluorescence by single cell imaging.

Numerous inhibitors of Chk1 with distinct chemotypes have been described in the literature. HT29 cells were treated with 1 μM V158411 [33], 3 μM LY2603618 [38], 3 μM MK-8776 [39], 3 μM GNE-900 [40] or 0.3 μM ARRY-1A [41] for 24 hours and the fraction of γH2AX, pRPA32 (S4/S8) or pChk1 (S317) positive nuclei determined. Two distinct classes of Chk1 inhibitors were identified; those that induced a strong increase in all three markers (including V158411, LY2603618 and ARRY-1A) and those that did not (including MK-8776 and GNE-900, Figure 9A). This was not due to a failure to inhibit Chk1 as all of the inhibitors decreased Chk1 autophosphorylation by >95 % (Figure 9B), potentiated gemcitabine cytotoxicity in HT29 cells (Supplementary Figures S10A and S10B) and, in combination with gemcitabine, altered Chk1-depdendent biomarkers in a predictive fashion (Supplementary Figure S10C). Live cell imaging was utilized to understand the effect of the Chk1 inhibitors on HT29 cell proliferation. V158411, LY2603618 and ARRY-1A rapidly stopped cell proliferation within 24 hours of inhibitor addition at concentrations that increased γH2AX, pRPA32 (S4/S8) or pChk1 (S317) staining. In comparison, MK-8776 and GNE-900 appeared to slow rather than stop cell proliferation in HT29 cells and produced growth response curves distinct to the other three inhibitors (Figure 9C). Analysis of the cell cycle distribution, following 72 hour treatment, identified differences in cell cycle distribution between the two classes. V158411, LY2603618 or ARRY-1A treatment dramatically decreased the G1 fraction of cells and increased the fraction of cells with a DNA content equivalent to G2/M or >G2/M (Figure 9D). MK-8776 and GNE-900 decreased the G1 fraction of cells and increased the fraction of cells with a DNA content equivalent to G2/M or >G2/M but to a much lesser extent than that observed with the other three Chk1 inhibitors.

In summary, we have extensively characterized the cellular response to single-agent Chk1 inhibition by V158411. This has provided some novel observations including (i) Chk1 inhibition by V158411 induced DNA damage in S-phase cells that was dose- and time-dependent; (ii) induction of γH2AX correlated with activation of ATR, ATM and DNA-PKcs signaling; (iii) γH2AX appearance preceded ssDNA or RPA exhaustion and was negative for TUNEL-staining; (iv) complete and sustained Chk1 kinase inhibition was necessary; (v) correlation between increased γH2AX and growth inhibition with the mechanism of growth inhibition being cell line dependent and included apoptosis, mitotic slippage and DNA damage-induced senescence; (vi) γH2AX and cleaved caspase-3 expression were mutually exclusive events; and (vii) two classes of Chk1 inhibitor were identified – those that induce γH2AX, pChk1(S317) and pRPA32 (S4/S8), and those that do not.

## DISCUSSION

The clinical development of Chk1 inhibitors has focused primarily on their ability to potentiate the cytotoxicity of DNA damaging chemotherapy drugs. There is a growing realization, however, that Chk1 inhibitors may possess anti-tumor efficacy when administered as a single agent with pre-clinical anti-tumor activity observed in a range of cancer models [25, 28, 30, 34, 35].

Previous work has demonstrated an involvement for Chk1 in maintaining replication fork progression [42, 43], controlling replication initiation [44-46] and protecting cells from DNA damage arising from replication fork collapse [47, 48]. Knockdown of Chk1 increased DNA replication initiation, phosphorylation of ATR targets and DNA breakage [49]. The accumulation of DNA damage was dependent on Cdc25A [50]. Similar results were obtained with UCN-01 [49]. UCN-01 is a non-specific pan-kinase inhibitor derived from staurosporine and effects induced by this molecule cannot be reliably attributed to Chk1 inhibition [51]. Here we demonstrate that a selective inhibitor of Chk1 induces DNA damage in S-phase cells and this correlates with phosphorylation of ATR, ATM and DNA-PKcs targets. DNA damage occurred rapidly (within 2 hours) following V158411 administration with staining appearing pan-nuclear rather than as foci commonly observed with DNA damaging agents. The presence of nuclei with pan-nuclear γH2AX staining that were TUNEL negative is intriguing. This was not associated with apoptosis (as has previously been demonstrated [52]) as expression of γH2AX and cleaved caspase-3 was mutually exclusive in cells treated with V158411. Pan-nuclear H2AX phosphorylation can occur in the absence of DSBs. Ectopic ATR activation can induce pan-nuclear γH2AX in the absence of DNA damage [53] as can changes to chromatin structure [54]. Phosphorylation of H2AX at undamaged chromatin is mediated by ATM and DNA-PKcs [55] with ATM activated by alterations in chromatin structure in the absence of DSBs [56]. Almost complete inhibition of Chk1 kinase activity was necessary to induce a robust increase in γH2AX staining and inhibition of cell proliferation. This was in direct contrast to Chk1 inhibition in combination with a DNA damaging agent such as gemcitabine or irinotecan where a much lower concentration of V158411 increased γH2AX [32].

Inhibition of ATR results in unscheduled origin firing leading to ssDNA accumulation, global exhaustion of RPA and replication catastrophe in a fraction of early S-phase cells [36, 57]. Chk1 inhibition by V158411 induced DNA damage prior to ssDNA accumulation and RPA exhaustion. LY2606368, a structurally unrelated Chk1 inhibitor, depleted the pool of RPA2 and induced DNA damage with similar kinetics to V158411 [58]. The work of Buisson *et al* [57] suggests there are distinct roles for ATR and Chk1 in countering S-phase replication stress. ATR inhibitors kill cells under high replication stress and Chk1 and DNA-PK mediate a backup pathway to suppress origin firing. This results in a lower threshold for replication stress induced cell death for a Chk1 inhibitor than an ATR inhibitor.

Inhibition of Chk1 in the four tumor cell lines examined resulted in persistent DNA damage for at least 72 hours after the addition of V158411. Persistent DNA damage, especially in telomeres, can result in irreversible cell cycle arrest; a process referred to as DNA damage-induced senescence [59, 60]. The response to persistent Chk1 inhibitor induced DNA damage was cell line dependent. A2058, HT29 and MDA-MB-231 cells underwent permanent cell cycle arrest with little caspase-dependent apoptosis. U2OS cells underwent significant caspase-dependent apoptosis following Chk1 inhibition that was mutually exclusive with γH2AX expression. U2OS was the only cell line of the four studied with functional p53. Further work is required to understand the role of p53 in tumor cell responses to Chk1 inhibition. The anti-proliferative activity of V158411 may be further accentuated due to the effective abrogation of S and G2/M DNA damage checkpoints [61] and homologous recombination repair, both processes controlled by Chk1 [21, 62]. Abrogation of cell cycle checkpoints allowed cells harboring replication-induced DSBs to exit into mitosis. In A2058, HT29 and MDA-MB-231 cell lines, cells with a DNA content >G2/M were observed that appeared to have failed cytokinesis potentially as a result of entering mitosis with high levels of unrepaired DNA damage.

Distinct differences in cellular responses were observed between the five different Chk1 inhibitors tested. Two groups of compounds could be clearly identified: those that induced DNA damage and ATR/ATM/DNA-PKcs activation at concentrations that concomitantly inhibited cellular proliferation (including V158411, LY2603618 and ARRY-1A) and those that did not (including MK-8776 and GNE-900). These differences could not be accounted for by differences in selectivity for Chk2. LY2603618, MK-8776, GNE-900 and ARRY-1A exhibit selectivity for Chk1 over Chk2 at the enzyme level and whilst V158411 exhibits little Chk1/Chk2 selectivity at the enzyme level, around 19-fold Chk1 selectivity was observed at the cellular level. CDK2 activity is required for origin firing and its activity is suppressed by Chk1 [63]. Inhibition of Chk1 results in increased CDK2-mediated origin firing and replication stress. Inhibition of CDK2 reduces Chk1 inhibitor induced-γH2AX expression due to reduced origin firing and replication fork speed [29, 58, 64]. Differences in CDK2 selectivity exist between the five Chk1 inhibitors evaluated. LY2603618 and V158411 [33, 38] are highly selective for Chk1 over CDK2 (CDK2 IC_50_ > 20 μM) as is ARRY-1A (>100-fold selective for Chk1 versus CDK2) [41]. However, MK-8776 and GNE-900 exhibit CDK2 inhibitory activity at the concentrations tested (IC_50_ 0.16 and 0.37 μM respectively) [39, 40]. This reduced CDK2 selectivity potentially explains why these compounds exhibit a Chk1 mode-of-action at lower concentrations of inhibitor in combination with a DNA damaging cytotoxic but not at the higher concentrations required for single agent activity.

We extensively used high content single cell immunofluorescent microscopy and live cell imaging to evaluate the tumor cell response to V158411. Understanding the temporal and spatial response of tumor cells to V158411 will further aid clinical development of this compound especially for selecting appropriate dosing regimens, biomarker discovery and clinical implementation, identifying sensitive patient populations, and determining potential novel drug combinations.

## MATERIALS AND METHODS

### Cell lines and cell culture

Cell lines were purchased from the American Type Culture Collection (ATCC, LGC Standards, Teddington, UK), established as a low passage cell bank and then routinely passaged in our laboratory for less than 3 months after resuscitation. These were routinely cultured in media containing 10 % FCS and 1 % penicillin/streptomycin (complete media) at 37°C in a normal humidified atmosphere supplemented with 5 % CO_2_. Cells were authenticated by STR profiling (LGC Standards).

### Compounds

V158411 was from Vernalis Research. LY2603618 and MK-8776 were purchased from Selleckchem (Houston, USA), and GNE-900 and ARRY-1A were synthesized in house according to published information. All were prepared as 20 mM DMSO stocks. Compounds were serially diluted in the appropriate solvent to 500x or 1000x then to 5x or 10x in complete media before addition to cells to yield a 1x final concentration.

### Antibodies

Antibodies against Chk1, pChk1 (S317), pChk2 (T68), pChk2 (S516), pH2AX (S139), pCdc2 (Y15), pHH3 (S10), GAPDH, DNA-PKcs, pDNA-PKcs (S2056), and cleaved Caspase-3 were purchased from Cell Signaling Technologies (Danvers, USA); pChk1 (S296), RPA32, RPA70 and BrdU (clone MoBu-1) from Abcam (Cambridge, UK); pRPA32 (S4/S8) from Bethyl Laboratories (Montgomery, USA) and pH2AX (S139) (clone JBW301) from Merck Millipore (Watford, UK). Antibodies were used at the manufacturer's recommended dilutions.

### Immunoblotting

Cells were washed once with PBS and lysed in RIPA buffer containing protease and phosphatase inhibitor cocktails (Sigma, Poole, UK). Protein concentration was determined using a BCA kit (ThermoScientific, Hemel Hempstead, UK). Equal amounts of lysate were separated by SDS-PAGE and western blot analysis conducted using the antibodies indicated above. ImageJ software (NIH) was used for densitometric analysis.

### Single cell immunofluorescent imaging

Following compound treatment, cells were fixed in 3.7 % paraformaldehyde (in PBS) at room temperature for 15 minutes, washed with PBS, blocked with 5% normal goat serum in 0.3 % Triton X100 in PBS for 1 hour at room temperature and then incubated with primary antibody diluted in antibody dilution buffer (1 % BSA, 0.3 % Triton X100 in PBS) at 4°C for 16 hours. Cells were washed with PBS then incubated with an Alexa-labelled secondary antibody (1:500, Life Technologies, Inchinnan, UK) and Hoechst 33342 (1 μg/ml) in antibody dilution buffer at room temperature for 60 minutes. Following washing with PBS, cells were imaged with an Operetta high content imaging system (Perkin Elmer, Sear Green, UK) at 10x or 20x magnifications and analyzed using Harmony software (Perkin Elmer). Typically 3 to 5 fields per well were imaged which equated to between approximately 1000 and 4000 cells/well.

### RPA70 loading

At the end of the treatment period, cells were washed once with PBS then pre-extracted with ice cold 0.2 % Triton X100 in PBS on ice for 1 minute. Cells were fixed and RPA70 and γH2AX detected as described above.

### Detection of ssDNA

To visualize ssDNA, cells were cultured in 10 μM BrdU for 42 hours before treatment with V158411. BrdU present in ssDNA was detected by single cell immunofluorescent image analysis using the Operetta high content imaging system with a specific monoclonal antibody under non-denaturing conditions.

### TUNEL assay

TUNEL-positive cells were detected using a click-iT^®^ TUNEL Alexa Fluor imaging assay (Life Technologies). Labelled cells were subsequently detected with the Operetta high content imager.

### High content cell cycle analysis

High content cell cycle analysis was conducted essentially as previously described [65]. For DNA-only analysis, cells were fixed and permeabilzed with 3.7 % paraformaldehyde/0.3 % Triton X100 in PBS at room temperature for 15 minutes. Cells were washed twice in PBS then stained with Hoechst 33342 (1 μg/ml) in PBS at room temperature for 30 minutes.

For multiparametric cell cycle analysis, cells were labelled with 10μM EdU for 15 minutes immediately prior to fixation with 3.7 % paraformaldehyde in PBS at room temperature for 15 minutes. Cells were washed twice in PBS then twice in 3 % BSA in PBS before permeabilization with 0.5 % Triton X100 in PBS for 20 minutes at room temperature. Cells were washed twice with 3 % BSA in PBS before incorporated EdU was labelled with an Alexa Click-iT EdU labeling kit (Life Technologies). Following blocking, for 30 minutes with 5 % normal goat serum in PBS, cells were incubated with an anti-pHH3 (S10) primary antibody diluted in antibody dilution buffer at 4°C for 16 hours. Cells were washed with PBS then incubated with an Alexa-labelled secondary antibody (1:500, Life Technologies) and Hoechst 33342 (1 μg/ml) in antibody dilution buffer at room temperature for 60 minutes. Following washing with PBS, cells were imaged with an Operetta high content imaging system (Perkin Elmer), at 10x magnification and analyzed using Harmony software (Perkin Elmer).

### Apoptosis

Cleaved caspase-3 (CC3) was detected in fixed cells using a monoclonal antibody to the amino-terminal residues adjacent to Asp175. Caspase-3/7 activity in live cells was determined using CellEvent caspase-3/7 green ReadyProbes reagent (Life Technologies). Cells growing in FluoroBrite media (Life Technologies) were seeded in 96-well CellCarrier plates (Perkin Elmer) and allowed to attach for 24 hours before addition of compound and CellEvent reagent. Images were acquired as indicated using the Alexa488 fluorescence channel and digital phase imaging modalities on the Operetta high content imaging system at 10x magnification. Temperature was maintained at 37° C and CO_2_ at 5 % with the live cell chamber module.

### Cell proliferation assay

5000 cells per well were seeded in 96-well plates and incubated overnight. Cells were treated with a 10-point titration of compound for 72 hours. The effect on cell proliferation was determined with sulphorhodamine B (SRB) after fixation with 10% trichloroacetic acid and read on a Victor plate reader (Perkin Elmer). GI_50_ values were calculated in Microsoft EXCEL using an XLFit software add-in (ID Business Solutions, Guildford, UK).

### High content live cell imaging

Cells were seeded in 96-well CellCarrier plates and allowed to attach for 24 hours before addition of compound. Images were acquired using the brightfield and digital phase imaging modalities on the Operetta high content imaging system at 10x magnification. Cell confluency was determined from the brightfield images using the ‘Find Texture Regions’ building block coupled with PhenoLOGIC texture-based segmentation in the Harmony software. Cell number was determined by analysis of the digital phase images with the ‘Find Cells’ building block in Harmony.

## SUPPLEMENTARY MATERIALS FIGURES AND TABLES


