# Supplementary material for: Inhibition of Chk1 with the small molecule inhibitor V158411 induces DNA damage and cell death in an unperturbed S-phase
Source: Oncotarget. 2016 Nov 4;7(51):85033–48. doi: 10.18632/oncotarget.13119 (PMC5356717; doi:10.18632/oncotarget.13119)
Supplement: Supplementary file 1 [file oncotarget-07-85033-s001.pdf]

Inhibition of Chk1 with the small molecule inhibitor V158411 induces DNA damage and cell death in an unperturbed S-phase

SUPPLEMENTARY MATERIALS

REFERENCE

1. Sousa FG, Matuo R, Tang SW, Rajapakse VN, Luna A, Sander C, Varma S, Simon PH, Doroshow JH, Reinhold WC, Pommier Y. Alterations of DNA repair genes in the NCI-60 cell lines and their predictive value for anticancer drug activity. DNA Repair (Amst). 2015;28: 107-15.

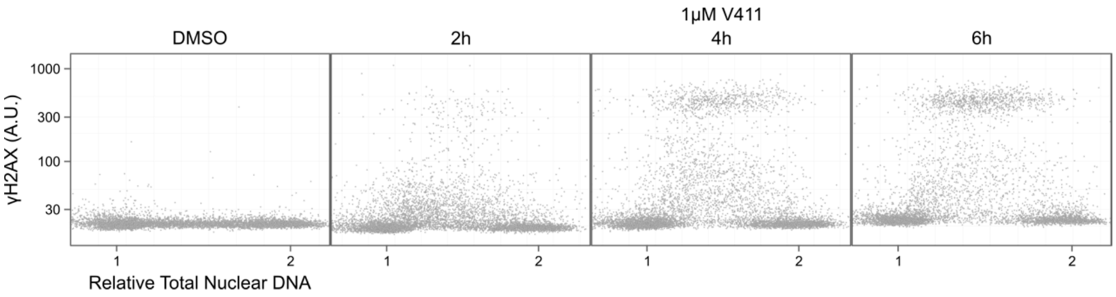

Supplementary Figure S1: Related to figure 2. U2OS cells were treated with 1 μM V411 for 2 to 6 h. Total nuclear intensity of DNA and γH2AX was determined by single cell immunofluorescent imaging.

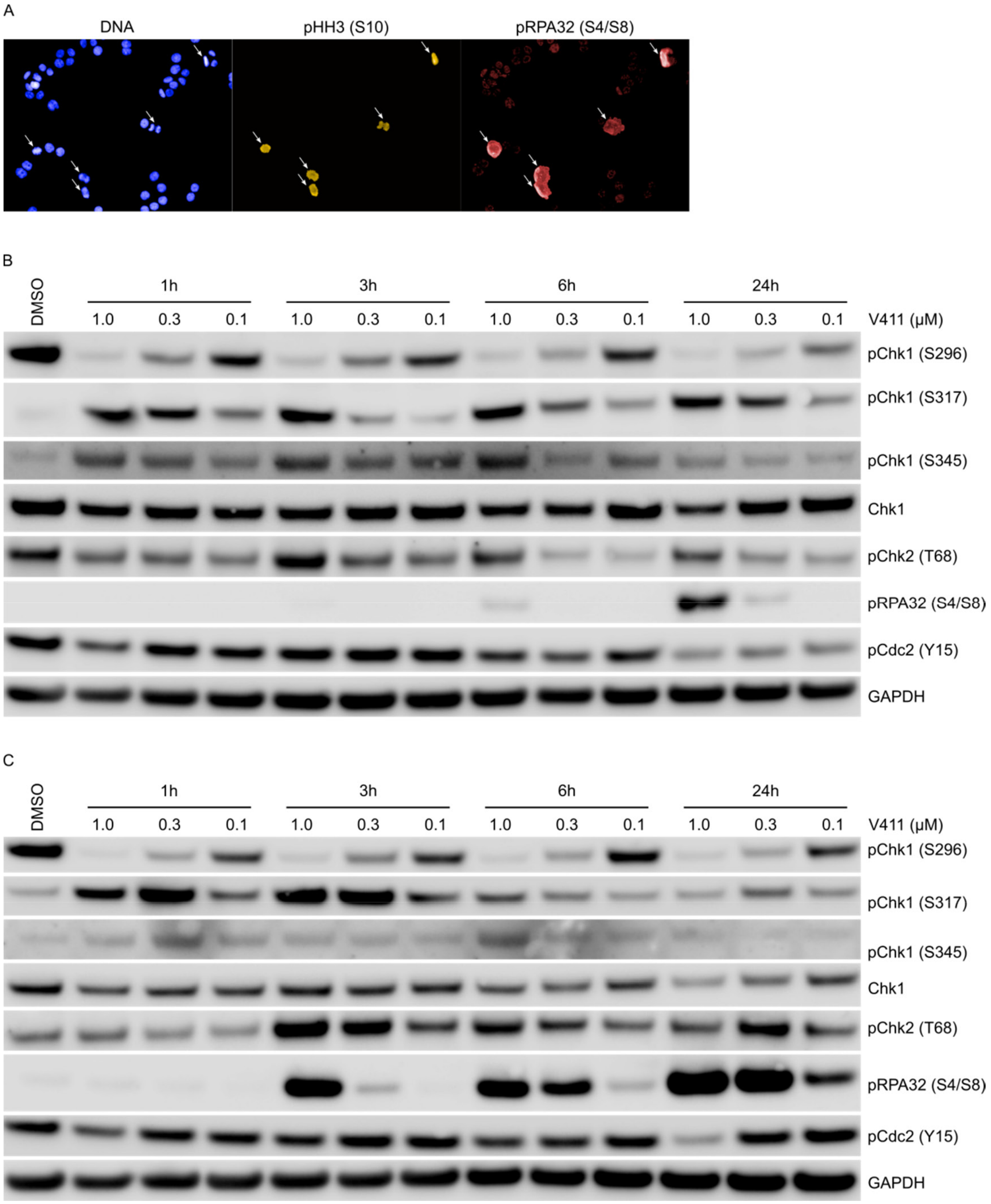

Supplementary Figure S2: Related to figure 3. A. Example images of untreated HT29 cells fixed and stained for DNA, pHH3 (S10) and pRPA32 (S4/S8). Arrows indicate mitotic cells. HT29 **B.** or U2OS **C.** cells were treated with the indicated concentrations of V411 for 1 to 24 h. Cell lysates were immunoblotted with the indicated antibodies.

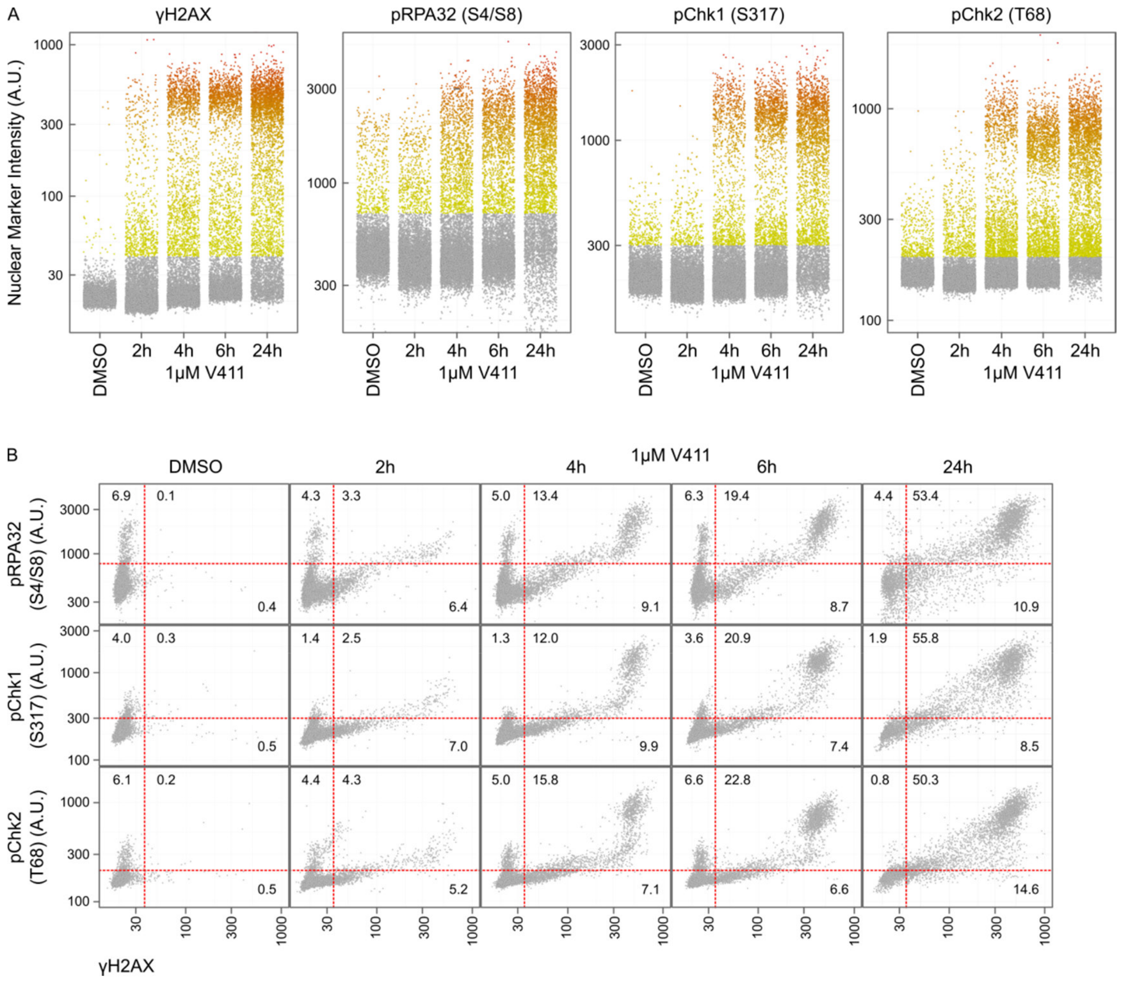

Supplementary Figure S3: Related to figure 4. A. The time-dependent increase in average nuclear γH2AX, pRPA32 (S4/S8), pChk1 (S317) and pChk2 (T68) intensity in U2OS cells following treatment with 1 μM V411 was determined by single cell immunofluorescent imaging. **B.** Co-expression analysis of γH2AX with pRPA32 (S4/S8), pChk1 (S317) and pChk2 (T68) in U2OS cells. The numbers indicate the fraction of positive cells in each quadrant.

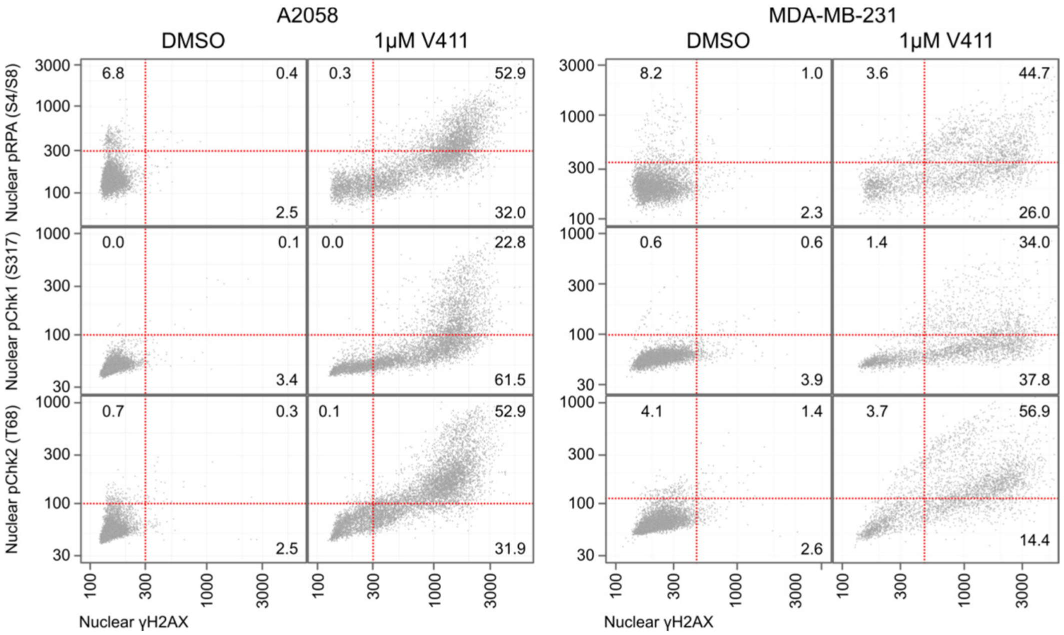

Supplementary Figure S4: Related to figure 4. Co-expression analysis of γH2AX with pRPA32 (S4/S8), pChk1 (S317) and pChk2 (T68) in A2058 or MDA-MB-231 cells following 24 h treatment with 1 µM V411. The numbers indicate the fraction of positive cells in each quadrant.

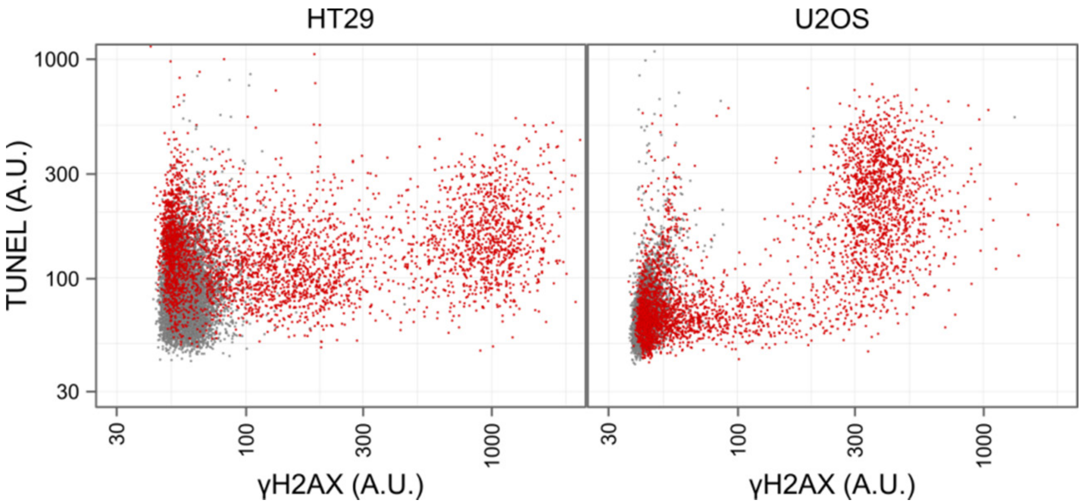

Supplementary Figure S5: Related to figure 5. HT29 or U2OS cells were treated with DMSO (grey) or 1 μM V411 (red) for 6 h. Cells were probed with an anti-γH2AX antibody and TUNEL stained with a Click-iT TUNEL assay kit. Single cell mean nuclear fluorescence intensity was assessed by imaging.

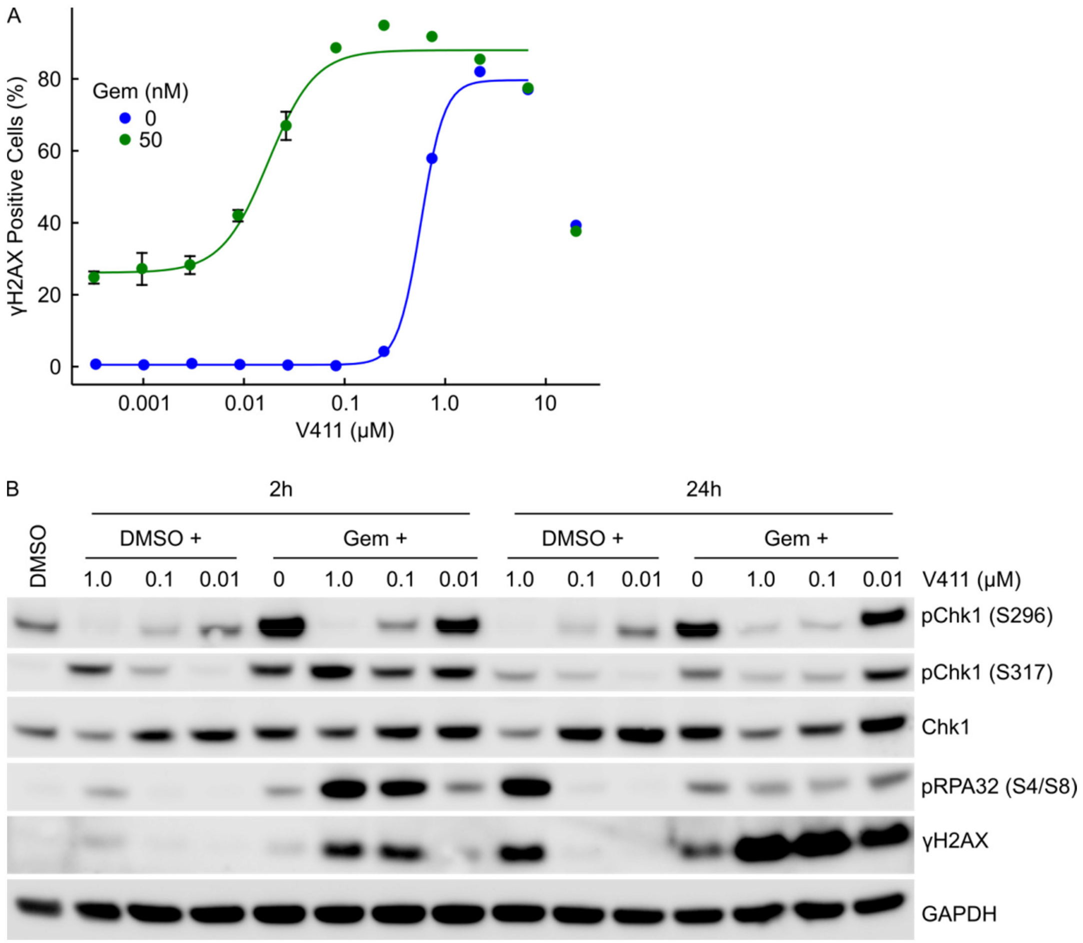

Supplementary Figure S6: Related to figure 6. A. HT29 cells were treated with increasing concentrations of V411 in combination with 0 or 50 nM gemcitabine (Gem) for 24 h. The fraction of γH2AX positive cells was determined by single cell immunofluorescent imaging (n=4, mean ± SD). **B.** HT29 cells were treated with 0 or 50 nM gemcitabine (Gem) for 16 h followed by the indicated concentrations of V411 for 2 or 24 h. Cell lysates were immunoblotted with the indicated antibodies.

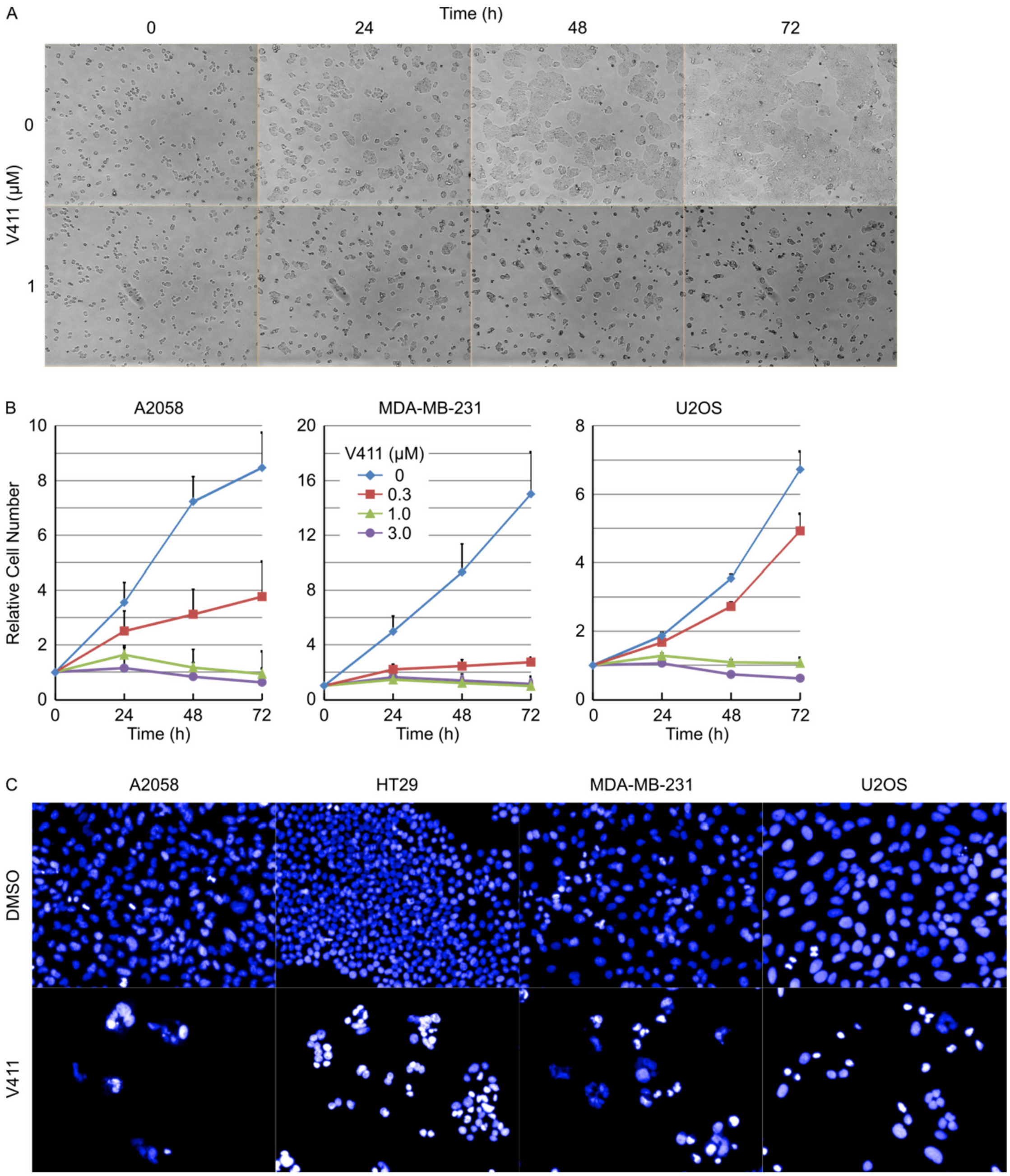

Supplementary Figure S7: Related to figure 7. A. Example brightfield images of HT29 cells used to determine cellular confluency. **B.** Cell lines were treated with the indicated concentrations of V411 and cellular number determined by repeated live cell imaging using a digital phase imaging algorithm (n=6, mean ± SD). Note due to their morphology, the determination of single cell boundaries by DPC was not possible for HT29 cells. **C.** Example images of cells fixed and stained with Hoechst to determine cell cycle distribution.

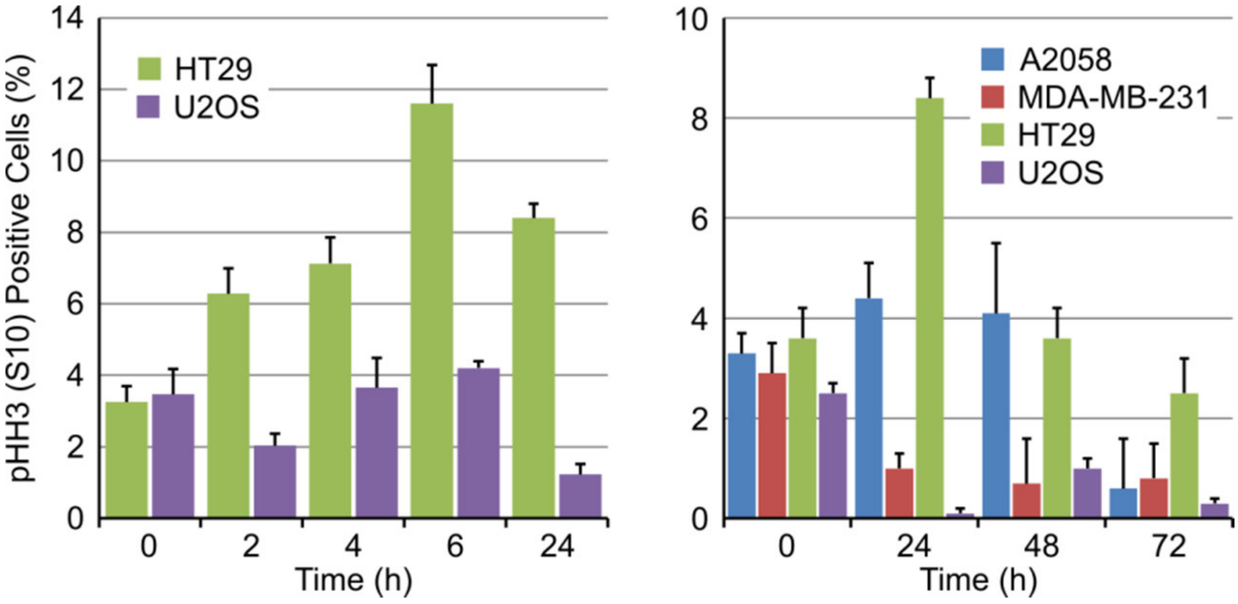

Supplementary Figure S8: Related to figure 7. Cells were treated for the indicated times with 1 μM V411 and the fraction of pHH3 (S10) positive nuclei was determined by single cell immunofluorescent analysis (n=4, mean ± SD).

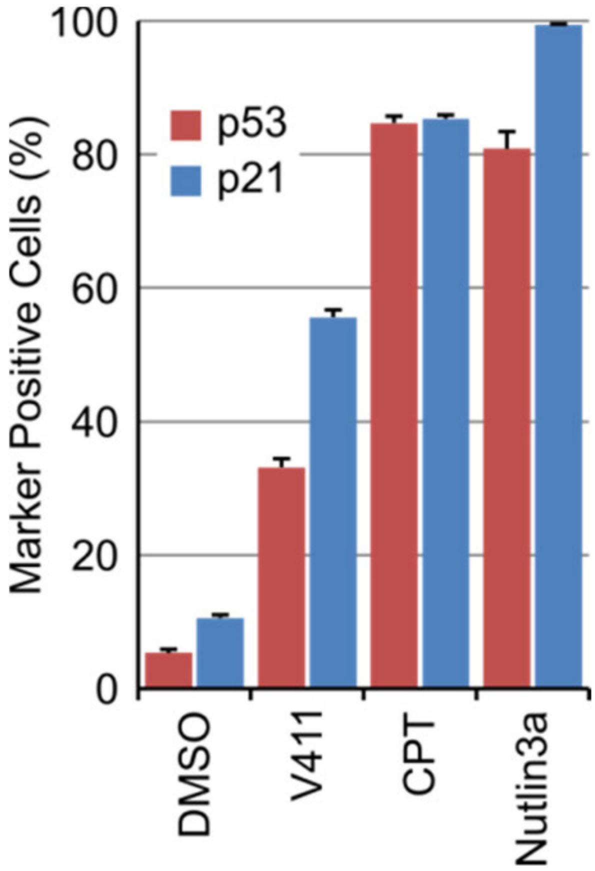

Supplementary Figure S9: Related to figure 8. U2OS cells were treated with 1 µM V411, 1 µM camptothecin (CPT) or 5 µM Nutlin3a for 24 h. The fraction of p53 or p21 positive nuclei was determined by single cell immunofluorescence analysis (n=4, mean ± SD).

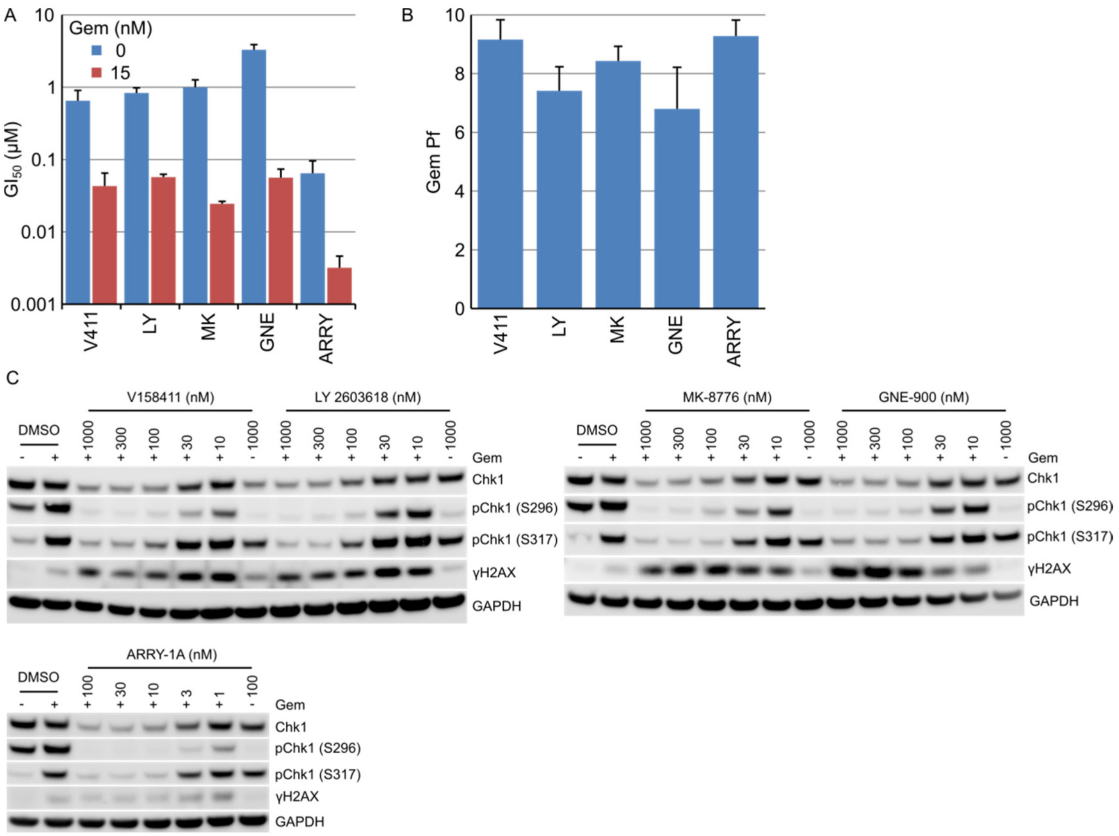

Supplementary Figure S10: Related to figure 9. A. HT29 cells were treated with increasing concentrations of the indicated Chk1 inhibitors in the presence of 0 or 15 nM gemcitabine for 72 h. Cells were fixed, stained with SRB and the GI_{50} determined using XLFit (n ≥ 3, mean ± SD). **B.** HT29 cells were treated with increasing concentrations of gemcitabine in combination with DMSO, 0.3 µM V411, LY, MK or GNE, or 0.03 µM ARRY for 72h. Cells were fixed, stained with SRB and the GI_{50} determined using XLFit (n ≥ 3, mean ± SD). The potentiation factor (Pf) was calculated as the gemcitabine GI_{50} DMSO/gemcitabine GI_{50} Chk1i. **C.** HT29 cells were treated with 50 nM gemcitabine for 18 h followed by the indicated concentrations of Chk1 inhibitors for a further 24 h. Cell lysates were immunoblotted with the indicated antibodies.

Supplementary Table S1: Characteristics of cell lines used in study

Cell Line	Tumor Etiology	Mutations in Cancer Gene Census
A2058	Melanoma	BRAF, CCNB1IP1, CTCF, LIFR, MAP2K1, MLLT1, MYO5A, PTEN, PTPRB, PTPRK, SF3B1, STK11, STRN, SYK, TET2, TP53
HT29	Colon	APC, BRAF, BRD3, CAMTA1, ETV6, FCRL4, KMT2C, PAX3, PIK3CA, PRDM16, SMAD4, TP53, TRIM24, WRN, ZFHX3
MDA-MB-231	Breast	ATM, AXIN2, BRAF, CD79A, CLTCL1, CRTC3, GAS7, KRAS, LONP1, NF1, NF2, PBRM1, PCSK7, PDGFRA, PER1, SLC45A3, TCEA1, TP53
U2OS	Osteosarcoma	CTNNB1, FAT1, LIFHR, SLC34A2, TET2, TSC2, ZFHX3

Cell line mutational data was extracted from the COSMIC Cell Lines Project at the Sanger Institute.

Supplementary Table S2: DNA repair (DNAR) gene functional alterations in cell lines used in study

Cell Line	DNAR Genes with		
	Homozygous Mutations	Heterozygous Mutations	Reduced Expression
A2058	TP53	ATR, DCLRE1C, MBD4, MSH5, PARP4, PTEN, SAMHD1	FANCM
HT29	TP53, WRN	ALKBH8, APC, FANCM, POLN, POLQ, SLFN11, XAB2	HLTF
MDA-MB-231	MSH3, PER1	ATM, MPG, PNKP, RECQL, RRM2, TP53	
U2OS		ALKBH1, PPM1D, SMC2	

The list of 260 DNAR genes analyzed came from [1]. Cell line mutational data was extracted from the COSMIC Cell Lines Project at the Sanger Institute and expression data from the Cancer Cell Line Encyclopedia at the Broad Institute.
